# Supplementary material for: Splice-Junction-Based Mapping of Alternative Isoforms in the Human Proteome
Source: Cell Rep. Author manuscript; Available in PMC 2020 Jan 15. (PMC6961840; doi:10.1016/j.celrep.2019.11.026)
Supplement: 2 [file NIHMS1546469-supplement-2.zip › DF1/PXD006675/RightAtrium/RightAtrium_23_TTN_SNCTVSVHVSEPATIVEKPESIK.pdf]

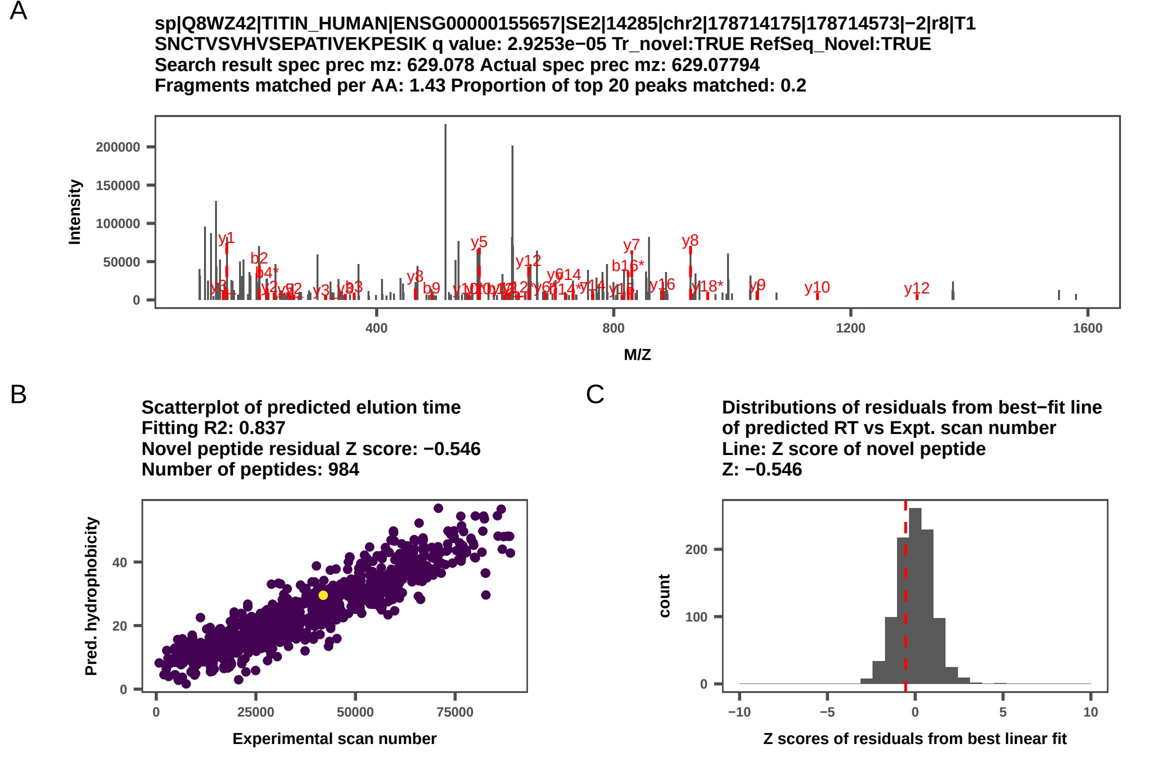
A
sp|Q8WZ42|TITIN_HUMAN|ENSG00000155657|SE2|14285|chr2|178714175|178714573|−2|r8|T1
SNCTVSVHVSEPATIVEKPESIK q value: 2.9253e−05 Tr_novel:TRUE RefSeq_Novel:TRUE
Search result spec prec mz: 629.078 Actual spec prec mz: 629.07794
Fragments matched per AA: 1.43 Proportion of top 20 peaks matched: 0.2
Intensity
200000
150000
100000
50000
0
y1
b2
b4*
y5
y12
y7
b16*
y8
y8
y6
14
y16
y18*
y9
y10
y12
b9
400
800
1200
1600
M/Z
B
Scatterplot of predicted elution time
Fitting R2: 0.837
Novel peptide residual Z score: −0.546
Number of peptides: 984
Pred. hydrophobicity
40
20
0
0
25000
50000
75000
Experimental scan number
C
Distributions of residuals from best−fit line
of predicted RT vs Expt. scan number
Line: Z score of novel peptide
Z: −0.546
count
200
100
0
−10
−5
0
5
10
Z scores of residuals from best linear fit
